# The body inversion effect in chimpanzees (*Pan troglodytes*)

**DOI:** 10.1371/journal.pone.0204131

**Published:** 2018-10-03

**Authors:** Jie Gao, Masaki Tomonaga

**Affiliations:** 1 Primate Research Institute, Kyoto University, Inuyama, Aichi, Japan; 2 Research Fellow of Japan Society for the Promotion of Science, Tokyo, Japan; Consiglio Nazionale delle Ricerche, ITALY

## Abstract

Bodies are important social cues for animals. Body recognition in humans is deteriorated by inversion. This inversion effect suggests the configural processing of bodies, which is different from the processing used for other objects. However, it is not known if this type of body processing exists in non-human primates. We tested seven chimpanzees using upright and inverted chimpanzee body stimuli and other stimuli in matching-to-sample tasks to examine the body inversion effect and the body parts that invoke it. Our results reflected the body inversion effect for intact chimpanzee bodies, bodies with complete body contours, and bodies with clear faces but not for the objects and other conditions that did not present complete body contours and clear faces. The results show that chimpanzees share configural body processing with humans and that bodies are special to them compared with other objects. The results also revealed the functions of faces and body contours in configural processing by chimpanzees.

## Introduction

Both faces and bodies provide important social cues for animals. Previous studies have reported the inversion effect for faces widely, in humans (e.g. [1–3]) and non-human primates (e.g. [4–6]). The performance of face recognition decreases significantly when faces are inverted compared to when they are shown upright. This is called the inversion effect, and it has been regarded as a solid index for configural processing (reviewed in [7]). This type of processing is different from the way used to process other objects, featural processing, where no inversion effect is shown (e.g. [1–3]). Studies using event-related potentials (ERPs) have found that the N170 is larger for faces than other objects, and that inverted faces cause delayed and amplified N170 compared to upright faces, while other objects do not have this effect [8, 9]. In functional magnetic resonance imaging (fMRI) studies, face stimuli activate certain brain areas (e.g. the fusiform face area in the lateral fusiform gyrus) more than other objects do, and the inversion of faces affects this activation [10, 11]. These findings suggest that faces are special to humans and some non-human animals compared to other objects. Diamond and Carey [12] proposed that humans use configural processing to the objects that they have expertise with. Also, the parts of these objects, including faces, have the same arrangements, which are called first-order relations; their variations from a standard sample are called second-order relations. Researchers have hypothesized that configural processing might have evolved to let animals detect social cues according to a pre-set template, allowing such cues to be processed more quickly, for faces and other targets that require expertise to deal with [8].

Bodies, while obviously different from faces, are also very important in animals’ lives. Bodies are the direct agents with which animals explore and interact with the environment. They are the basis of embodied recognition and self-recognition, enabling the individual to distinguish between self and others [13, 14]. Bodies are directly related to performing and understanding others’ physical activities. Chimpanzees, for example, use their bodies to forage for food, manipulate tools, and perform other daily activities [15]. The bodies of other individuals are important social cues. Chimpanzees are able to recognise other individuals based on their bodies (e.g. [16, 17]), learn to use tools by viewing others’ actions [15], and use various kinds of bodily gestures for social communication [18, 19]. Bodies also provide very important social cues, and may actually be more frequently used than faces, especially for animals that live in dense forest environments where they cannot see each other’s faces easily. Also, bodies share the relations that are similar to the first-order and the second-order relations in faces: bodies of the same species all share the same structure and at the same time vary in detailed parts with different individuals. It is therefore reasonable to hypothesise that animals also process bodies in a special way that is different from the way they process other objects. At the same time, bodies are totally different from faces in terms of both appearance and function, so it is unclear whether configural processing is used to process bodies or not.

Reed et al. [20] first reported the body inversion effect in human participants, and their findings were later supported by other studies [21, 22]. Humans’ performance of body perception decreases when bodies are inverted, compared to when they are upright. This suggests that humans process bodies configurally, and that bodies are also special compared with inanimate objects. Additionally, studies using ERP showed an N170 similar to that evoked by faces when body stimuli were presented to humans, and delayed N170 when bodies were inverted [23, 24]. Studies using fMRI have found locations in the fusiform gyrus selectively activated by body stimuli, and this fusiform body area is adjacent to, and partially overlapping with, the fusiform face area [23, 25]. These findings suggest that humans employ specific processing for bodies.

However, little is known about the evolution of body processing, or its occurrence in non-human primates. Matsuno and Fujita [26] have found the body inversion effect for human 3D models in capuchin monkeys. Previous studies in macaques have also reported that cells in the anterior part of the superior temporal sulcus (STS) are specifically activated by body postures and body motion [23, 27–29], and the body selective area is very close to the face selective area in the STS [23, 30]. These findings suggest that non-human primates may have a special way to process bodies compared to other objects. However, no behavioural data (i.e. the inversion effect) has been reported, so little is known about the specific processing of bodies if, indeed, there is any. For example, it is unclear whether bodies are subject to configural processing in the same way that faces are. In capuchin monkeys, however, it is still unknown whether the inversion effect extends to the bodies of conspecifics. Configural body processing could facilitate the detection of bodies, and it is reasonable to infer that for social primates in their natural environment it is more important to detect the bodies of conspecifics than it is for other species.

This study tested whether chimpanzees (*Pan troglodytes*) exhibit the body inversion effect in response to their conspecifics. Chimpanzees, which are the closest relatives to humans, share many common features with humans and, unlike monkeys, chimpanzees have a fusiform area (the area which in humans has been found to contain both the fusiform face area and the fusiform body area [10, 11, 23, 25]). Chimpanzees, like humans, are highly social. They recognise other individuals based on their faces and bodies [13, 14], and use bodily gestures to communicate [15, 16]. The close evolutionary relationship between the two species may have led to a shared approach to body processing. At the same time, there are also dramatic differences between the two species related to the functions of the body, and these may have led to differences in how they process bodies. For example, in many places, chimpanzees live in heavily forested areas (e.g. [31]), whereas early humans lived in savannah environments (e.g. [32]). Moreover, the two species show different body postures when they move, as humans are bipedal and chimpanzees are quadrupedal. These differences in living environments and anatomy cause chimpanzees to exhibit more inverted and diverse body postures than humans, and this may render chimpanzees less focused on orientation, which, in turn, could diminish the body inversion effect [33].

Apart from examining the body inversion itself, it is also important to understand the function of body parts in configural processing. In humans, it has been found that the face part is an important cue for the body inversion effect [22]. This implies that body discrimination relies heavily on the facial part, and that body configural processing and face configural processing may have different mechanisms, even though they are both configural. In this study, we manipulated body stimuli to test which body parts are important for body recognition, and to examine in detail the mechanism of body configural processing in chimpanzees.

**Fig 1:**
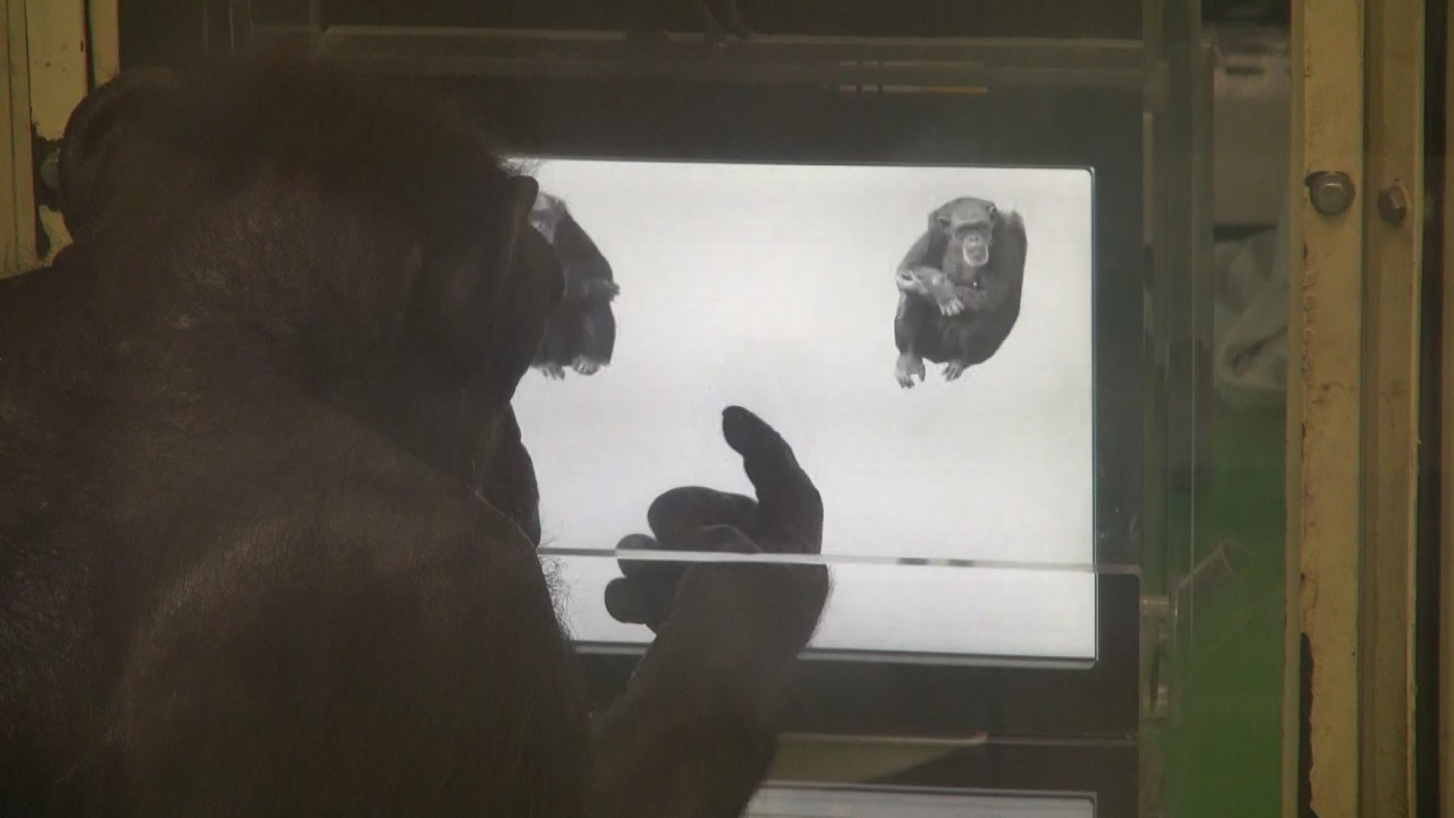
A demonstration of chimpanzee Ai performing the task (two choice alternatives). Chimpanzees used a touch screen. When they made a correct choice, a food reward was delivered by the feeder via a tube.

**Fig 2:**
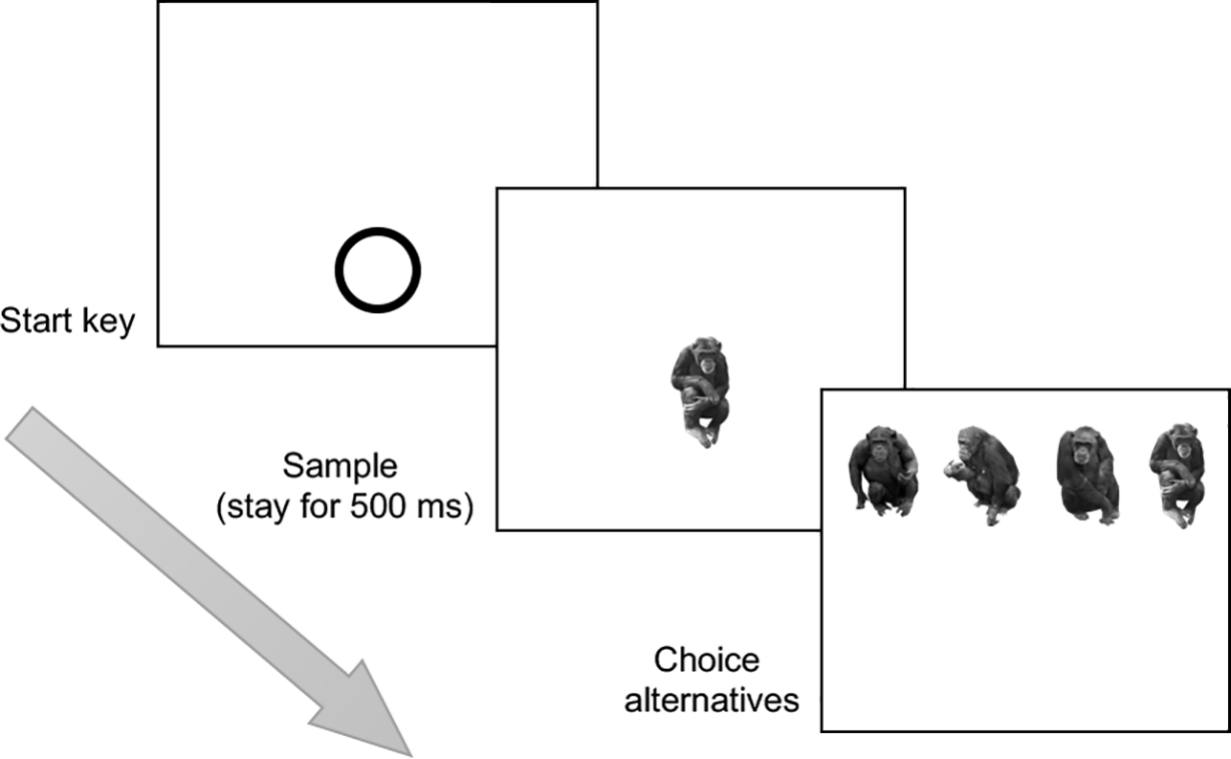
An example trial (four choice alternatives). The figure shows one upright trial under the intact body condition. First, a start key appeared on the screen. This was followed by the appearance of the sample, which disappeared when the chimpanzee touched it after remaining on the screen for 500 ms, to be replaced by a set number of alternatives. Here, one trial with four choices is shown.

**Fig 3:**
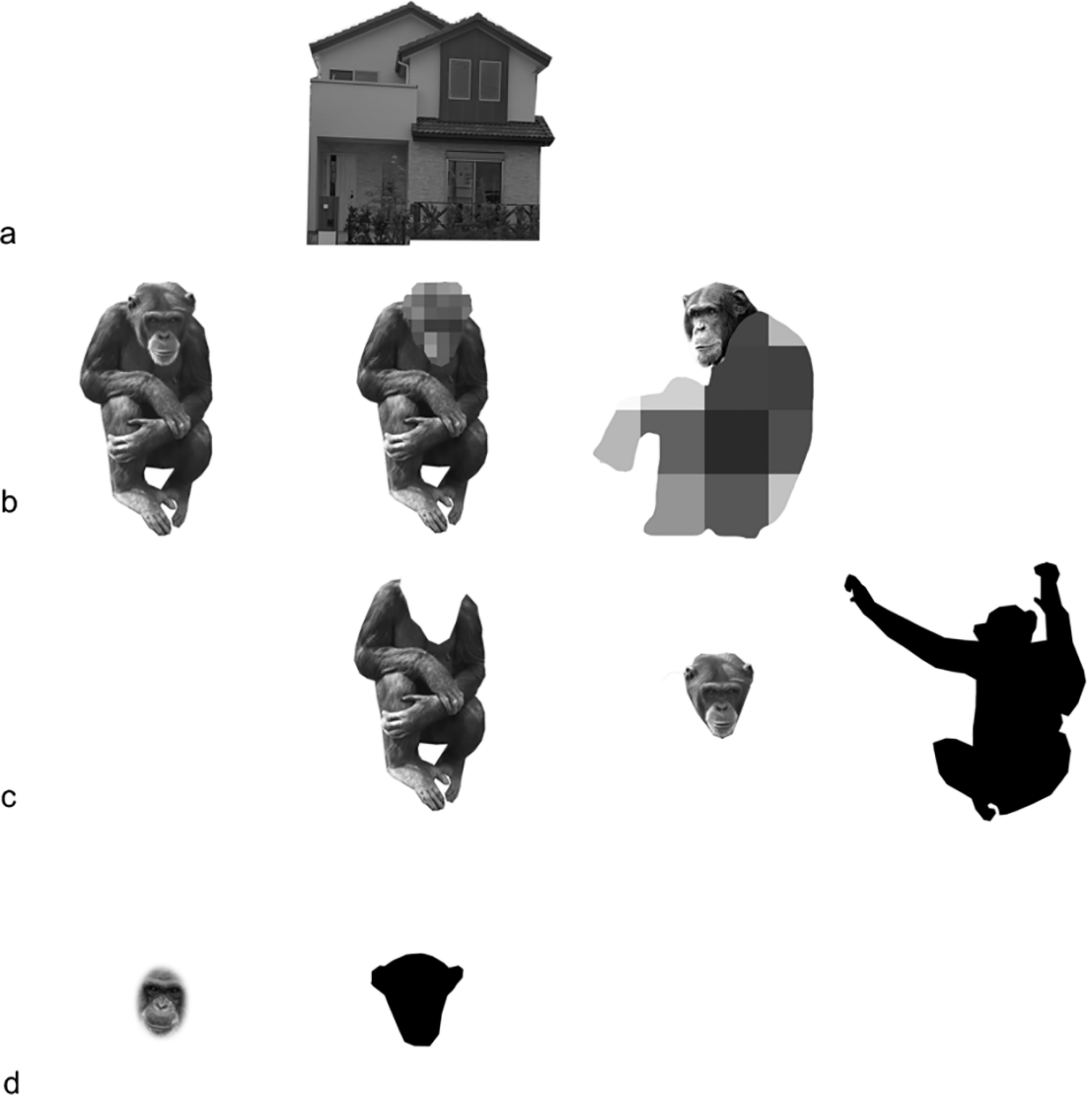
Examples of stimuli used in the experiments. **a)** Examples of stimuli from Experiment 1 (from left to right): intact chimpanzee body; house. **b)** Examples of stimuli from Experiment 2a (from left to right): intact chimpanzee body; body only (mosaic face); face only (mosaic body part). **c)** Examples of stimuli from Experiment 2b (from left to right): intact body; body only (without face); face only (without body part); body silhouette. **d)** Examples of stimuli from Experiment 3 (from left to right): face without contour; face silhouette.

To understand the evolutionary aspects of body processing, this study investigated body processing in chimpanzees by examining the body inversion effect. We also performed a detailed examination of the parts that could be significant cues for the body inversion effect in chimpanzees. Seven chimpanzees participated in Experiment 1a, and six participated in Experiments 1b–3 (Table 1). Participants engaged in zero-delayed matching-to-sample tasks (Figs 1 and 2) with trials of upright stimuli and trials of inverted stimuli mixed together. Experiment 1 included two conditions: 1) the intact-chimpanzee-body condition and 2) the house condition. If the chimpanzees showed the inversion effect in the body condition and not in the house condition, that would suggest that they use configural body processing, and that it is different from the way in which they process other objects. Experiment 2a included three conditions: 1) the intact-body condition as a positive control; 2) the only-body-clear condition, under which the face was blurred with a mosaic pattern, and the rest of the body was clearly visible; 3) the only-face-clear condition, under which only the face was clearly visible, and the rest of the chimpanzee body was blurred with a mosaic pattern. Experiment 2b included four conditions: 1) the intact-body condition as a positive control; 2) the only-body condition, under which chimpanzee bodies without faces were presented; 3) the only-face condition, under which only chimpanzee faces were presented; and 4) the silhouette condition, which involved chimpanzee silhouettes. If chimpanzees failed to exhibit the inversion effect in some of these conditions, it would suggest that the missing parts in those conditions are important in the evocation of the inversion effect. Experiment 3 re-examined the face inversion effect in chimpanzees (Fig 3). We compared the error rates and response times of the upright and inverted trials under each condition to examine the inversion effect.

**Table 1:** General characteristics of the seven chimpanzees.

Name	GAIN ID Number*	Sex	Age (when the study started)	Kinship	Participated in
Ai	0434	Female	40	Ayumu’s mother	Experiments 1–3
Ayumu	0608	Male	16	Ai’s son; Pal’s sibling	Experiments 1–3
Chloe	0441	Female	36	Cleo’s mother	Experiments 1–3
Cleo	0609	Female	16	Chloe’s daughter	Experiments 1–3
Pan	0440	Female	33	Pal’s mother	Experiment 1a
Pal	0611	Female	16	Pan’s daughter; Ayumu’s sibling	Experiments 1–3
Pendesa	0095	Female	39	N.A.	Experiments 1–3

Note

*Identification number for each chimpanzee listed in the database of the Great Ape Information Network (GAIN); https://shigen.nig.ac.jp/gain/

## Methods

### Participants

Seven chimpanzees (six of whom also participated in Experiments 1b–3) participated in Experiment 1a (Table 1). The participants belonged to two social groups, containing a total of 12 individuals, which are housed at the Primate Research Institute, Kyoto University (KUPRI; Inuyama, Aichi, Japan). All participants were born in captivity except for Ai, who was brought to KUPRI from the wild when she was about one year old (details are available in the Great Ape Information Network, see Table 1). Their living environment includes an outdoor compound (700 m^2^) and attached indoor compounds [16]. From their living facilities, the chimpanzees have views of many houses in the city of Inuyama, from different angles. All chimpanzees had full access to food and water during the study. The chimpanzees had previous experience with cognitive tasks involving touch screens, including activities related to numerical sequence learning, short-term memory, acquisition of an artificial language, visual attention and visual search, and facial recognition [5, 33–36].

All procedures adhered to the Japanese Act on the Welfare and Management of Animals. The daily care and use of the chimpanzees adhered to the 2010 Guidelines for the Care and Use of Laboratory Primates of KUPRI. The research proposal was approved by the Animal Welfare and Animal Care Committee of KUPRI and also by the Animal Research Committee of Kyoto University.

### Apparatus

The participants sat in an experimental booth and performed tasks on a touch screen with a 15-inch LCD display (1,024 × 768 pixels, 1 pixel = 0.297 mm). Chimpanzees faced the screen head-on; screens were not tilted significantly in any direction (Fig 1). During the experiments, the chimpanzees could move freely, but they would always sit in front of the screen about 40 cm away. They always kept their postures natural and relaxed, and heads upright. When the chimpanzees made the correct choice in a trial, a piece of apple or raisin was provided via a feeder in conjunction with a chime sound. When they made a wrong choice, an error buzzer sounded, and no food was provided. The food reward was delivered by a universal feeder via a tube to a food tray placed at the bottom of the display. All experimental events were controlled by a computer. The experimental programs were written and operated with Microsoft^®^ Visual Basic^®^ 2010 software (Microsoft Corp.; Redmond, WA, USA).

### Stimuli (Fig 3)

Each experiment contained two or more conditions (e.g. intact body, house, etc.), and each condition was repeated for eight sessions. Each session included 40 trials: 20 were upright trials, in which all stimuli had the upright orientation, and the other 20 were inverted trials, in which all stimuli were inverted. The upright and inverted trials were mixed, and the order was randomly determined. Each condition included 40 pictures: 20 were in the upright position, and the remaining 20 were identical to the first 20 but in the inverted position (i.e. 40 pictures in total). All pictures were in black and white, and balanced in terms of luminance. Pictures of bodies and houses were about 400 * 400 px. Face stimuli presented in Experiment 3 were about 80 (width) * 115 (height) px, similar to faces presented in other conditions. In each trial, one stimulus served as the sample, and another stimulus (Experiment 1a) or three other stimuli (Experiments 1b, 2a, 2b, 3) was/were randomly chosen from those with the same orientation. The correct stimuli and their locations were counterbalanced in the experiment. The original pictures were manipulated using Adobe Photoshop software (Adobe Systems; San Jose, CA, USA) and Pixelmator (Pixelmator Team Ltd.; Vilnius, Lithuania). Some pictures were obtained from the Internet, and the rest were provided by Kumamoto Sanctuary, Wildlife Research Center of Kyoto University. We evaluated the similarity of all the pictures (more than the desired number) before choosing those used in the experiment. The participants were not familiar with the specific chimpanzees or houses in the stimulus pictures.

Both Experiment 1a and Experiment 1b had two conditions: 1) intact body and 2) house (Fig 3A). The house condition was a control condition [3, 5, 21]. In Experiment 2a, there were three conditions: 1) the intact-body condition, which was used as the control; 2) the only-body-clear condition, in which the face was replaced by a mosaic pattern; and 3) the only-face-clear condition, in which the body, with the exception of the face, was replaced by a mosaic pattern (Fig 3B). In Experiment 2b, there were four conditions: 1) the intact-body condition, which was used as a control; 2) the only-body condition, in which there were no faces; 3) the only-face condition, in which only the faces were shown; and 4) the body-silhouette condition, in which intact chimpanzee bodies were solid black (Fig 3C). In Experiment 3, there were two conditions: 1) the face-without-contour condition and 2) the face-silhouette condition (Fig 3D). In the face-without-contour condition, the outline of the face was replaced by an oval shape of 75 (width) * 110 (height) px, and only the face contents remained. Under the face-silhouette condition, the original contours remained but were filled with solid black.

### General procedure

A zero-delayed matching-to-sample task (Fig 2) was used. Experiment 1a offered two choices per trial; the other experiments offered four choices per trial. In each trial, the start key (a circle) initially appeared at the bottom centre of the touch screen against a white background. After the chimpanzees touched the start key, the sample stimulus appeared at the centre of the screen. The sample remained for 500 ms, then disappeared upon touching. At the same time, image choices appeared side by side in the top part of the screen. A correct choice was one in which the chimpanzees chose the image that was the same as the sample. Correct choices were followed by a piece of food as a reward accompanied by a chime sound. Otherwise, a buzzer would sound and no food would be presented. The inter-trial-interval (ITI) was 1.5 s, and the timeout was 2 s.

### Data analyses

Accuracy and response-time data were recorded and analysed. We compared data to determine whether the error rate (100%—accuracy) and the response times differed between all upright trials and all inverted trials under each condition. According to previous studies (e.g. [6]), if either the error rate or the response time were higher in the inverted trials than in the upright trials, an inversion effect would be deemed to be present. Data were analysed using a generalised linear mixed model (GLMM) in R 3.3.1 [37] using the *lme4* package [38]. We calculated the error rates of the 20 upright trials and the 20 inverted trials on a session-by-session basis. The distribution of the error rate was binomial. The fixed effect was orientation (upright or inverted). The random effects were participant ID and session number. Response time refers to the duration of time from the chimpanzee touching the sample to when s/he chose any one of the alternatives. Only the response times from correct trials were included in the analyses. We calculated the average response time of all correct upright trials and all correct inverted trials on a session-by-session basis. During the experiment, unexpected sounds from outside the experimental booths were sometimes heard, and the chimpanzees were interrupted; therefore, we initially discarded response times that were longer than the mean value plus 3 SDs. We then calculated the average of the remaining data points and used them for further analyses. The response-time data were normally distributed. We compared the null model and the model with the fixed-effect orientation via analysis of variance (ANOVA) using the *anova()* function to examine whether there were differences in the response times for the two orientations. Both models included the random effects of participant ID and session number.

## Results and discussion

### Results of Experiment 1a

#### Intact-body condition

**Fig 4:**
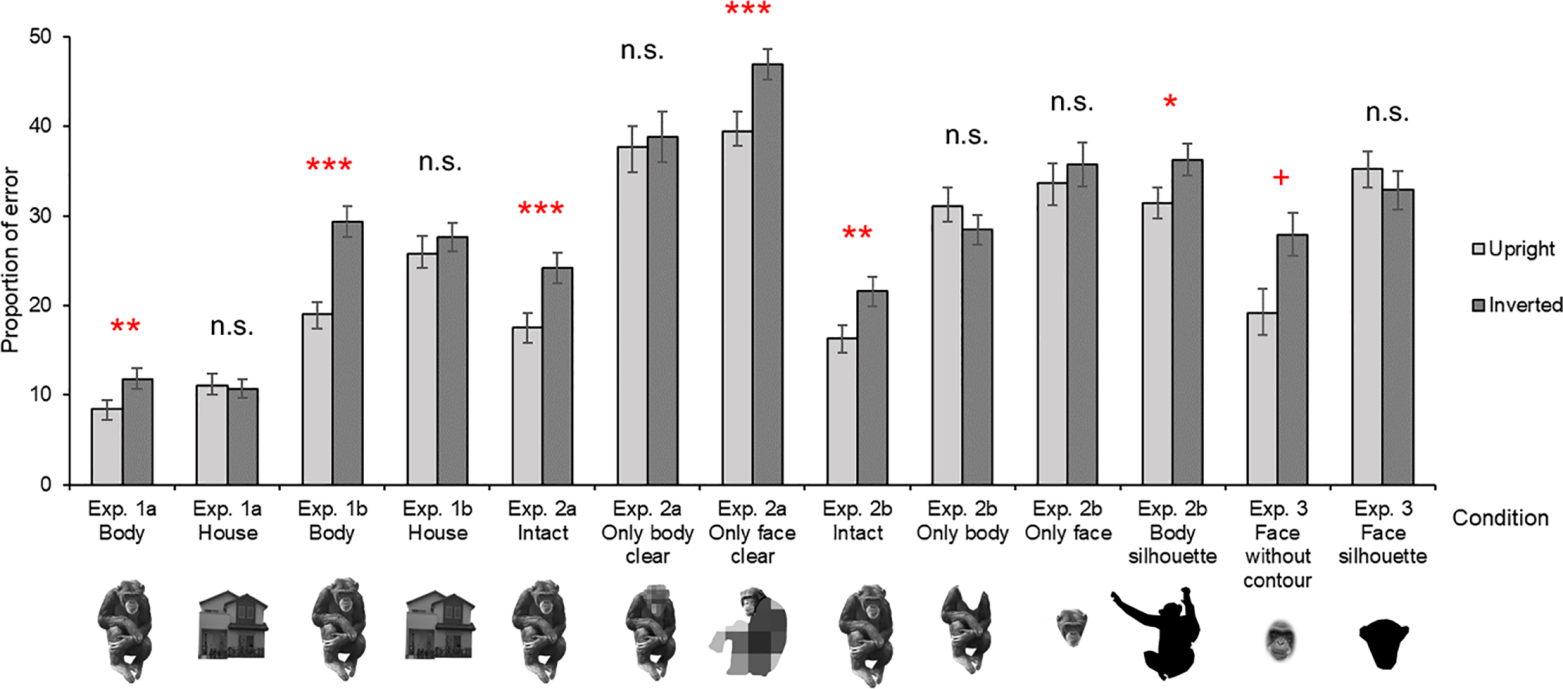
Proportions of errors in upright and inverted trials under each condition. Exp.: Experiment; n.s.: Not significant; + *p* < 0.07; * *p* < 0.05; ** *p* < 0.01; *** *p* < 0.001; Error bar: SEM.

The mean error rate in the upright trials was 8.393 ± 1.074%, and the mean error rate in inverted trials was 11.786 ± 1.185% (Fig 4). Generalised linear mixed model (GLMM) analyses showed the error rate was significantly higher in inverted than in upright trials (estimate of the fixed effect, orientation: -0.393; standard error: 0.144; z value = -2.726; *p* = 0.006; estimate of the intercept: -2.199). The variances of the random effects, participant ID and session number were 0.028 and 0.492, respectively, and their standard deviations (SDs) were 0.166 and 0.702, respectively.

#### House condition

The mean error rate in upright trials was 11.071 ± 1.272%, and the mean error rate in inverted trials was 10.714 ± 1.046% (Fig 4). GLMM analyses showed no significant difference between the inverted and the upright trials (estimate of the fixed effect, orientation: 0.038; standard error: 0.137; *z* value = 0.275; *p* = 0.783; estimate of the intercept: -2.233). The variances of the random effects, participant ID and session number, were 0.320 and 0, respectively, and their SDs were 0.566 and 0, respectively.

We found no significant differences between the response times in the upright and inverted trials under either condition (S1 Fig).

### Results of Experiment 1b

#### Intact-body condition

The mean error rate in upright trials was 19.063 ± 1.345%, and the mean error rate in inverted trials was 29.375 ± 1.701% (Fig 4). GLMM analyses showed the error rate was significantly higher in the inverted than in the upright trials (estimate of the fixed effect, orientation: -0.576; standard error: 0.109; *z* value = -5.278; *p* < 0.001; estimate of the intercept: -0.893). The variances of the random effects, participant ID and session number, were 0.077 and 0, respectively, and their SDs were 0.277 and 0, respectively.

#### House condition

The mean error rate in upright trials was 25.833 ± 1.891%, and that in inverted trials was 27.604 ± 1.596% (Fig 4). GLMM analyses showed no significant difference between the inverted and the upright trials (estimate of the fixed effect, orientation: -0.093; standard error: 0.105; *z* value = -0.891; *p* = 0.373; estimate of the intercept: -1.013). The variances of the random effects, participant ID and session number, were 0.205 and 0.011, respectively, and their SDs were 0.452 and 0.107, respectively.

We found no significant differences between the response times in the upright and inverted trials under either condition (S1 Fig).

### Discussion of Experiment 1a and 1b

In both Experiment 1a and Experiment 1b, the data on error rates reflected the inversion effect under the intact-body conditions but not under the house conditions. This body inversion effect suggests configural body processing, which differs from the featural strategy used to process other objects, including houses. This result suggests that chimpanzees and humans may adopt the same way of body processing, i.e. configural, and that, compared to other non-body/non-face objects, bodies, like faces, may also be special to chimpanzees.

In the following experiments, we examined the functions of different body parts in the inversion effect, and included the intact condition as a positive control.

### Results of Experiment 2a

#### Intact-body condition

The mean error rate in upright trials was 17.500 ± 1.644%, and the mean error rate in inverted trials was 24.167 ± 1.706% (Fig 4). GLMM analyses showed the error rate was significantly higher in the inverted than in the upright trials (estimate of the fixed effect, orientation: -0.421; standard error: 0.115; *z* value = -3.652; *p* < 0.001; estimate of the intercept: -1.191). The variances of the random effects, participant ID and session number, were 0.166 and 0.031, respectively, and their SDs were 0.407 and 0.177, respectively.

#### Only-body-clear condition

The mean error rate in upright trials was 37.708 ± 2.340%, and the mean error rate in inverted trials was 38.854 ± 2.830% (Fig 4). GLMM analyses showed no significant difference between the inverted and the upright trials (estimate of the fixed effect, orientation: -0.052; standard error: 0.097; *z* value = -0.537; *p* = 0.592; estimate of the intercept: -0.494). The variances of the random effects, participant ID and session number, were 0.301 and 0.067, respectively, and their SDs were 0.548 and 0.259, respectively.

#### Only-face-clear condition

The mean error rate in upright trials was 39.479 ± 2.112%, and the mean error rate in inverted trials was 46.875 ± 1.714% (Fig 4). GLMM analyses showed that the error rate was significantly higher in the inverted than in the upright trials (estimate of the fixed effect, orientation: -0.309; standard error: 0.094; *z* value = -3.306; *p* < 0.001; estimate of the intercept: -0.129). The variances of the random effects, participant ID and session number, were 0.088 and 0.016, respectively, and their SDs were 0.296 and 0.125, respectively.

No significant difference between the response times in the upright and inverted trials was found under any condition (S1 Fig).

### Discussion of Experiment 2a

The response times under the three conditions did not reflect an inversion effect. However, the error rates revealed an inversion effect under the intact-body condition and under the only-face-clear condition, but not under the only-body-clear condition.

The inversion effect under the intact-body condition was consistent with the results of Experiment 1, further supporting the robustness of the body inversion effect in chimpanzees and indicating that chimpanzees use configural body processing.

When the face was replaced with a mosaic pattern, no inversion effect was observed, which suggests that the face is an important cue for the inversion effect.

With the exception of the face, when body parts were replaced with a mosaic pattern, the inversion effect persisted. This suggests that the face and/or the body contour are/is very important for the inversion effect.

The results suggest that the face is an important cue for the inversion effect. However, a definitive conclusion about the function of body contours could not be drawn from this experiment. It is possible that an intact body contour or shape is also an important cue for the inversion effect, with the face being significant, which could explain why the inversion effect was observed under the only-face-clear condition but not under the only-body-clear condition. In Experiment 2b, we used the following conditions to examine the function of body contours in the body inversion effect: the intact-body condition; the only-body condition, under which the face was missing; the only-face condition; and the body-silhouette condition.

### Results of Experiment 2b

#### Intact-body condition

The mean error rate in upright trials was 16.354 ± 1.472%, and the mean error rate in inverted trials was 21.563 ± 1.639% (Fig 4). GLMM analyses showed the error rate was significantly higher in the inverted than in the upright trials (estimate of the fixed effect, orientation: -0.350; standard error: 0.119, *z* value = -2.948; *p* = 0.003; estimate of the intercept: -1.348). The variances of the random effects, participant ID and session number, were 0.196 and 0.008, respectively, and their SDs were 0.443 and 0.092, respectively.

#### Only-body condition

The mean error rate in upright trials was 31.042 ± 2.157%, and the mean error rate in inverted trials was 28.438 ± 1.659% (Fig 4). GLMM analyses showed no significant difference between the inverted trials and the upright trials (estimate of the fixed effect, orientation: 0.129; standard error: 0.102; *z* value = 1.271; *p* = 0.204; estimate of the intercept: -0.959). The variance and the SD of the random effects, participant ID, were 0.174 and 0.417, respectively, and those of the other random effects, session number, were both < 0.001.

#### Only-face condition

The mean error rate in upright trials was 33.646 ± 2.197%, and the mean error rate in inverted trials was 35.729 ± 2.410% (Fig 4). GLMM analyses showed no significant difference between the inverted and the upright trials (estimate of the fixed effect, orientation: -0.097; standard error: 0.099; *z* value = -0.986; *p* = 0.324; estimate of the intercept: -0.628). The variances of the random effects, participant ID and session number, were 0.247 and 0.036, respectively, and their SDs were 0.497 and 0.190, respectively.

#### Body-silhouette condition

The mean error rate in upright trials was 31.458 ± 1.736%, and the mean error rate in inverted trials was 36.250 ± 1.771% (Fig 4). GLMM analyses showed the error rate was significantly higher in the inverted than in the upright trials (estimate of the fixed effect, orientation: -0.219; standard error: 0.098; *z* value = -2.244; *p* = 0.025; estimate of the intercept: -0.579). The variances of the random effects, participant ID and session number, were 0.105 and 0.002, respectively, and their SDs were 0.324 and 0.041, respectively.

No significant difference between the response times in the upright and inverted trials was found under any condition (S1 Fig).

### Discussion of Experiment 2b

The response times did not reveal the inversion effect in any of the conditions. Based on the error rates, the inversion effect was present under the body-silhouette condition but not under the only-body or the only-face condition. This suggests that an intact body shape is needed for the inversion effect and for configural processing.

Taken together with the results of Experiment 2a, these data show that the inversion effect was present under the following conditions: 1) the intact-body conditions; 2) the only-face-clear condition in Experiment 2a (mosaic body); and 3) the body-silhouette condition in Experiment 2b. These three conditions all involved intact body shapes. Although the only-body-clear condition in Experiment 2a also contained intact body shapes (mosaic face), this condition did not show the inversion effect. Based on these results, we can conclude intact body contours are needed to elicit the inversion effect. Second, the body-silhouette condition may be considered special, probably because chimpanzees see black body “silhouettes” quite frequently in their everyday lives. Indeed, chimpanzees live in the forest and, in sunlight, they are able to see other individuals as black shapes from various angles and distances. This could explain the inversion effect under the silhouette condition. Third, under all the conditions that involved intact body contours, except for the silhouette condition, clear faces were needed to invoke the body inversion effect, and this may explain why only the only-body-clear condition (Experiment 2a; mosaic face) did not elicit the inversion effect. Chimpanzees may lose focus in the absence of a face and, as a result, their processing may change from a configural manner, leading to no inversion effect. In summary, combining the results from Experiment 2a and Experiment 2b, we conclude that faces and intact body contours are important cues for the body inversion effect—that is, for the configural processing of bodies.

Studies with human participants have found no inversion effect when the head was missing, indicating the significance of the face for configural processing [22]. This is similar to our finding in chimpanzees. This suggests that humans and chimpanzees might share similar mechanisms of configural body processing. At the same time, humans showed the inversion effect to human bodies with blurred faces [22], while this study suggests clear faces are needed to invoke the body inversion effect in chimpanzees. It indicates that the mechanisms of configural body processing might also show differences between the two species. More rigorous comparative studies are needed to draw detailed conclusions about shared processing mechanisms [7].

Another issue is that no inversion effect was found under the face-only condition in Experiment 2b, whereas previous research found the face inversion effect in chimpanzees [4–6]. This result is inconsistent with previous studies. Because the face stimuli under this condition were the same size as the faces under the intact-body condition, they were relatively smaller on the screen. Although they were still distinguishable from each other, their small size may have allowed chimpanzees to use the face contour as the cue to complete the task instead of attending to the content of the face, which would have invoked the face inversion effect [6]. In Experiment 3, we used face stimuli without contours and with contours only (face silhouette) to confirm the existence of the face inversion effect in chimpanzees.

### Results of Experiment 3

#### Face-without-contour condition

The mean error rate in upright trials was 39.583 ± 2.696%, and the mean error rate in inverted trials was 43.646 ± 2.431% (Fig 4). GLMM analyses showed the error rate was marginally significantly higher in the inverted than in the upright trials (estimate of the fixed effect, orientation: -0.182; standard error: 0.097; *z* value = -1.883; *p* = 0.060; estimate of the intercept: -0.268). The variances of the random effects, participant ID and session number, were 0.345 and 0.032, respectively, and their SDs were 0.588 and 0.178, respectively.

#### Face-silhouette condition

The mean error rate in upright trials was 46.563 ± 1.987%, and the mean error rate in inverted trials was 46.771 ± 2.124% (Fig 4). GLMM analyses showed no significant difference between the inverted and the upright trials (estimate of the fixed effect, orientation: -0.009; standard error: 0.094; *z* value = -0.094; *p* = 0.925; estimate of the intercept: -0.135). The variances of the random effects, participant ID and session number, were 0.191 and 0.006, respectively, and their SDs were 0.437 and 0.078, respectively.

No significant difference between the response times in the upright and inverted trials was found (S1 Fig).

### Discussion of Experiment 3

Although the response-time data did not show any inversion effect under either condition, the error rates reflected a marginally significant inversion effect under the face-without-contour condition but not under the face-silhouette condition.

These results tend to support the face inversion effect in chimpanzees, which is consistent with previous reports [4–6]. These data may also explain why the face inversion effect was not found in Experiment 2b; that is, probably due to the small size of the stimuli, chimpanzees may have paid attention only to the face contours instead of the face contents.

However, the absence of a face inversion effect in Experiment 2b does not invalidate our conclusions regarding the body inversion effect. The size of the face stimuli was small, but they were the same size and presented under the same conditions as the faces under the intact-body condition. Therefore, for purposes of control, it was highly reasonable to use these original face stimuli, and the conclusions should not be ignored despite the absence of the face inversion effect.

The absence of the face inversion effect under the face-silhouette condition also suggests that the face inversion effect and the body inversion effect, or the configural processing of faces and bodies, might rely on different underlying mechanisms [20, 22]. When configural processing is applied to bodies, the body contour is significant; however, the face contour is not important for faces. Furthermore, the presentation of only the face contour may interfere with configural processing. Although the configural processing of faces relies on the face contents, the removal of the eyes does not negatively affect this process (human study: [22]). However, the configural processing of bodies relies on the face part and the overall body shape, and the removal of the face violates these conditions (this study in chimpanzees; humans: [22]). These different characteristics imply that, although they are both configural and they both differ from other-object processing, face processing and body processing have different underlying mechanisms.

## General discussion

We report for the first time that chimpanzees show the body inversion effect. This suggests that they use configural body processing, which differs from the way other objects are processed. It shows that bodies are cognitively special to chimpanzees, in addition to being functionally special, compared with other objects.

Faces are also processed in a configural way, with the well-known face inversion effect, both in humans and non-human primates (e.g. [1–6]). In this study, we also revisited the face inversion effect in chimpanzees to explain the somewhat surprising results in Exp. 2b, the only-face condition. Combining the results from different conditions across the experiments, we could conclude that there is an interaction between face perception and body perception when the chimpanzees see bodies. There was no inversion effect in the only-body-clear condition in Exp. 2a, and in the only-face condition in Exp. 2b. If there is no interaction of face and body perception, then the simple combination of these two condition, i.e. intact bodies, would show no inversion effect. However, the results in intact-body conditions showed clear inversion effects. This indicates that the information in faces and other body parts interacts with each other to facilitate the recognition of bodies, thus showing the body inversion effect. The body inversion effect is not the face inversion effect, and it does not come from cues of single body parts.

Previous studies have found the body inversion effect in humans [20–22] and in capuchins [26]. This study found the inversion effect in chimpanzees. These results suggest that primate species might share configural body processing, as is the case with faces. As in the face inversion effect, the difference in the body postures of chimpanzees and humans does not interact with the body inversion effect [4–6]. Our data also imply that, despite many differences in their living environments, feeding behaviours, and other factors, chimpanzees and humans process bodies in the same way, which is different from how these species process other objects. However, detailed examinations are needed to draw reliable conclusions about the evolution of body processing. For example, as the above three species are highly social animals, it is not clear whether the body inversion effect is related to sociality. Additionally, shared configural processing does not necessarily mean that the mechanisms underlying this phenomenon are the same across species. Detailed investigations of various aspects of the body inversion effect are needed to understand the similarities and differences between these species in terms of the configural processing of bodies [7].

Configural processing serves important functions for animals. Indeed, it has been noted that expertise plays a role in invoking configural processing. Diamond and Carey [12] found that dog experts showed the inversion effect in response to dog stimuli, whereas non-experts did not. Rhodes et al. [39] found that people show more significant inversion effects to faces of the same race, which also suggests that familiarity has a role in configural processing. Gauthier and colleagues [40, 41] trained people to recognise “greebles”, a series of objects that share a first-order relationship, i.e. having the same arrangements of the parts. They found that greeble experts showed the inversion effect in response to greebles, and they also revealed a face-like N170 in ERP testing [42]. These results suggest that configural processing may occur for objects that frequently appear in our lives and require rapid and efficient detection [20]. On the one hand, configural processing developed during the evolutionary process for faces and bodies that are important to animals; on the other hand, this ability may be trained so it can be invoked for objects that require our expertise later in life. Bodies have been known to be functionally special as important social cues; this study showed, for the first time, that they are also cognitively special in non-human primates.

## Conclusions

In this study, we examined the body inversion effect in chimpanzees using computer-controlled tasks. We found that chimpanzees show the inversion effect for body stimuli but not for houses (Experiment 1). This suggests that they use configural processing, rather than the featural processing used for houses and other objects, in response to bodies. This indicates that bodies, like faces, are special compared to other (inanimate) objects. We also examined the functions of different body parts in configural processing. We found the face and the overall body contour are very important for the inversion effect (Experiment 2). This is the first study to report the body inversion effect in non-human primates. These findings suggest that bodies are processed in a special way by chimpanzees compared with other objects. It also suggests that non-human primates may process bodies in the same way as humans do, i.e. by means of configural body processing. Our results also emphasise the importance of face and body contour in configural body processing.

## Ethics

All procedures adhered to the Japanese Act on the Welfare and Management of Animals. The daily care and use of the chimpanzees adhered to the 2010 Guidelines for the Care and Use of Laboratory Primates of the Primate Research Institute, Kyoto University (KUPRI). The research proposal was approved by the Animal Research Committee of Kyoto University and also by the Animal Welfare and Animal Care Committee of KUPRI (#2016–064, #2017–106).

All participants were born in captivity except for Ai, who was brought to KUPRI from the wild when she was about one year old (details are available in the Great Ape Information Network, see Table 1). Their living environment includes an outdoor compound (700 m^2^) and attached indoor compounds [16]. From their living facilities, the chimpanzees have views of many houses in the city of Inuyama, from different angles. All chimpanzees had full access to food and water during the study. The chimpanzees had previous experience with cognitive tasks involving touch screens.

## Supporting information

S1 Fig Mean response times in upright and inverted trials under each condition. Exp.: Experiment; n.s.: Not significant; Error bar: SEM.
(TIF)

S1 Text Detailed results of the analyses of response time data. (DOC)

S1 Dataset Detailed session-by-session data. (XLS)
