# Supplementary figures and images for: The body inversion effect in chimpanzees (Pan troglodytes)
Source: PLoS One. 2018 Oct 3;13(10):e0204131. doi: 10.1371/journal.pone.0204131 (PMC6169876; doi:10.1371/journal.pone.0204131)

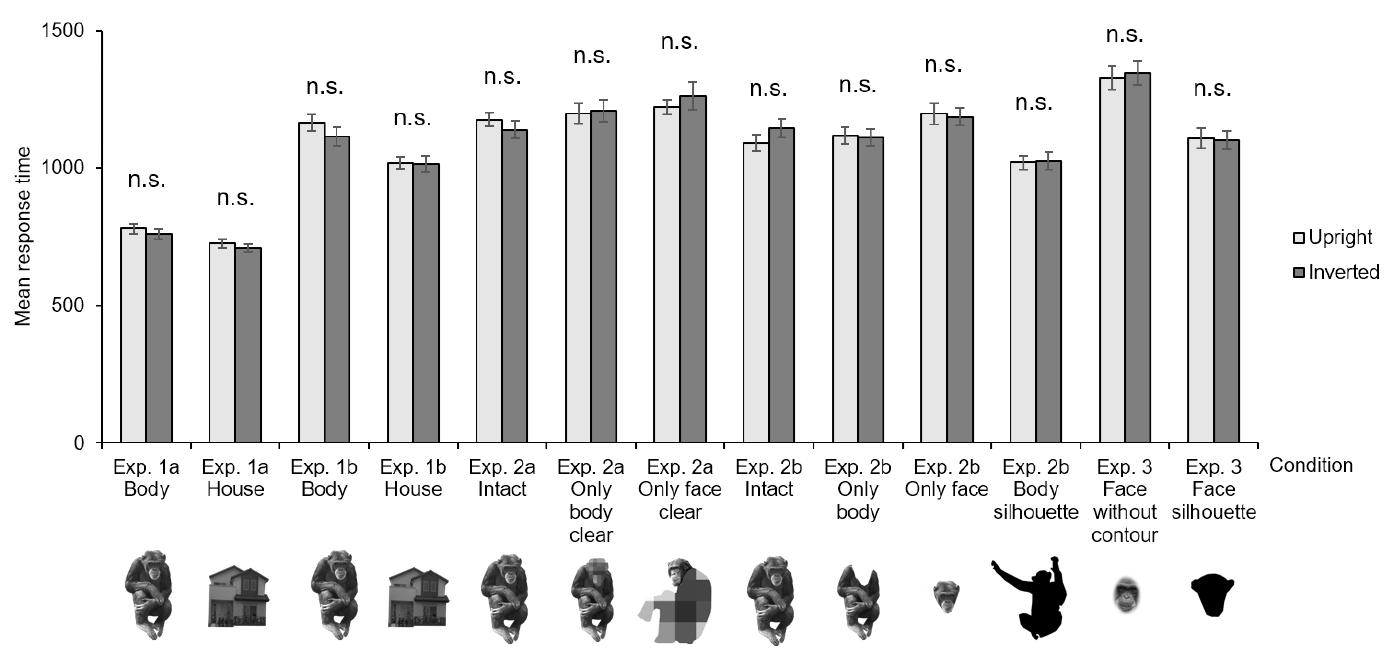

Supplement: S1 Fig — Exp.: Experiment; n.s.: Not significant; Error bar: SEM. (TIF) [file pone.0204131.s001.tif]
