# Supplementary material for: The body inversion effect in chimpanzees (Pan troglodytes)
Source: PLoS One. 2018 Oct 3;13(10):e0204131. doi: 10.1371/journal.pone.0204131 (PMC6169876; doi:10.1371/journal.pone.0204131)
Supplement: S1 Text — (DOC) [file pone.0204131.s002.doc]

Supporting Information

**The body inversion effect in chimpanzees (*Pan troglodytes*)**

Jie Gao^1, *^, Masaki Tomonaga^1^

1: Primate Research Institute, Kyoto University, Inuyama, Aichi, Japan, 484-8506

*Correspondence: gao.jie.87c@kyoto-u.jp

*PLOS ONE*

This supplementary information file contains detailed results of the analyses of response time in each condition (S1 Fig)

**Experiment 1a**

*Intact-body condition:* The mean response time in upright trials was 779 ± 17.9 s; the mean proportion of error in inverted trials was 758 ± 18.4 s. Generalized linear mixed model (GLMM) analyses showed no significant difference between the two orientations (*p* = 0.198).

*House condition:* The mean response time in upright trials was 725 ± 14.6 s; the mean response time in inverted trials was 709 ± 15.6 s. GLMM analyses showed no significant difference between the two orientations (*p* = 0.160).

**Experiment 1b**

*Intact-body condition:* The mean response time in upright trials was 1164 ± 31.5 s; the mean proportion of error in inverted trials was 1115 ± 33.5 s. GLMM analyses showed no significant difference between the two orientations (*p* = 0.0914).

*House condition:* The mean response time in upright trials was 1019 ± 22.3 s; the mean response time in inverted trials was 1014 ± 31.4 s. GLMM analyses showed no significant difference between the two orientations (*p* = 0.863).

**Experiment 2a**

*Intact-body condition:* The mean response time in upright trials was 1175 ± 25.7 s; the mean proportion of error in inverted trials was 1139 ± 32.1 s. GLMM analyses showed no significant difference between the two orientations (*p* = 0.123).

*Only-body-clear condition:* The mean response time in upright trials was 1198 ± 36.7 s; the mean response time in inverted trials was 1209 ± 40.9 s. GLMM analyses showed no significant difference between the two orientations (*p* = 0.784).

*Only-face-clear condition:* The mean response time in upright trials was 1221 ± 28.2 s; the mean response time in inverted trials was 1261 ± 49.6 s. GLMM analyses showed no significant difference between the two orientations (*p* = 0.358).

**Experiment 2b**

*Intact-body condition:* The mean response time in upright trials was 1092 ± 29.7 s; the mean proportion of error in inverted trials was 1145 ± 33.8 s. GLMM analyses showed no significant difference between the two orientations (*p* = 0.0721).

*Only-body condition:* The mean response time in upright trials was 1118 ± 31.3 s; the mean response time in inverted trials was 1111 ± 29.9 s. GLMM analyses showed no significant difference between the two orientations (*p* = 0.788).

*Only-face condition:* The mean response time in upright trials was 1197 ± 38.7 s; the mean response time in inverted trials was 1186 ± 32.5 s. GLMM analyses showed no significant difference between the two orientations (*p* = 0.660).

*Body-silhouette condition:* The mean response time in upright trials was 1020 ± 25.5 s; the mean response time in inverted trials was 1025 ± 31.8 s. GLMM analyses showed no significant difference between the two orientations (*p* = 0.878).

**Experiment 3**

*Face-without-contour condition:* The mean response time in upright trials was 1327 ± 42.7 s; the mean proportion of error in inverted trials was 1345 ± 42.5 s. GLMM analyses showed no significant difference between the two orientations (*p* = 0.616).

*Face-silhouette condition:* The mean response time in upright trials was 1108 ± 37.7 s; the mean response time in inverted trials was 1101 ± 33.3 s. GLMM analyses showed no significant difference between the two orientations (*p* = 0.815).
